# Effect of Different Adjuvants on Protection and Side-Effects Induced by *Helicobacter suis* Whole-Cell Lysate Vaccination

**DOI:** 10.1371/journal.pone.0131364

**Published:** 2015-06-26

**Authors:** Iris Bosschem, Jagadeesh Bayry, Ellen De Bruyne, Kim Van Deun, Annemieke Smet, Griet Vercauteren, Richard Ducatelle, Freddy Haesebrouck, Bram Flahou

**Affiliations:** 1 Department of Pathology, Bacteriology and Avian Diseases, Faculty of Veterinary Medicine, Ghent University, Merelbeke, Belgium; 2 Institut National de la Santé et de la Recherche Médicale, Unité 1138, Paris, France; 3 Centre de Recherche des Cordeliers, Equipe-Immunopathology and therapeutic immunointervention, Paris, France; 4 Sorbonne Universités, UPMC Univ Paris 06, UMR_S 1138, Paris, France; 5 Université Paris Descartes, Sorbonne Paris Cité, UMR_S 1138, Paris, France; Instituto Butantan, BRAZIL

## Abstract

*Helicobacter suis* (*H*. *suis*) is a widespread porcine gastric pathogen, which is also of zoonotic importance. The first goal of this study was to investigate the efficacy of several vaccine adjuvants (CpG-DNA, Curdlan, Freund’s Complete and Incomplete, Cholera toxin), administered either subcutaneously or intranasally along with *H*. *suis* whole-cell lysate, to protect against subsequent *H*. *suis* challenge in a BALB/c infection model. Subcutaneous immunization with Freund’s complete (FC)/lysate and intranasal immunization with Cholera toxin (CT)/lysate were shown to be the best options for vaccination against *H*. *suis*, as determined by the amount of colonizing *H*. *suis* bacteria in the stomach, although adverse effects such as post-immunization gastritis/pseudo-pyloric metaplasia and increased mortality were observed, respectively. Therefore, we decided to test alternative strategies, including sublingual vaccine administration, to reduce the unwanted side-effects. A CCR4 antagonist that transiently inhibits the migration of regulatory T cells was also included as a new adjuvant in this second study. Results confirmed that immunization with CT (intranasally or sublingually) is among the most effective vaccination protocols, but increased mortality was still observed. In the groups immunized subcutaneously with FC/lysate and CCR4 antagonist/lysate, a significant protection was observed. Compared to the FC/lysate immunized group, gastric pseudo-pyloric metaplasia was less severe or even absent in the CCR4 antagonist/lysate immunized group. In general, an inverse correlation was observed between IFN-γ, IL-4, IL-17, KC, MIP-2 and LIX mRNA expression and *H*. *suis* colonization density, whereas lower IL-10 expression levels were observed in partially protected animals.

## Introduction

*Helicobacter suis* (*H*. *suis*) is a Gram-negative, spiral-shaped bacterium and a worldwide spread pathogen colonizing the stomach of up to 90% of slaughter pigs. Even higher colonization rates are observed in adult boars and sows [1]. Infection with *H*. *suis* causes gastritis and a decrease in daily weight gain [2]. Although not always straightforward, several studies attribute a role to this pathogen in the development of gastric ulcer disease in pigs [2]. Economic losses due to the stomach ulcerations are believed to be substantial [3]. *H*. *suis* is also of zoonotic importance. Infection in human patients has been associated with gastritis, peptic ulceration and mucosa associated lymphoid tissue lymphoma [3].

Vaccination is considered to be a potentially valuable approach to control gastric *Helicobacter* infections and related disease development [4]. Besides the use of the appropriate antigen or combination of antigens, the choice of the immunization route and adjuvant play an important role in the outcome of vaccination studies. The use of an appropriate adjuvant has several benefits. Among other things, it reinforces the immune response, providing better and longer lasting protection against the pathogen. An adjuvant also allows the dose and dosing schedule of the antigen(s) to be decreased and modulated, reducing the cost and logistical complexity of administering vaccines [5]. Most *Helicobacter* vaccination strategies have been designed to generate an optimal immune response at the mucosal surface, in line with strategies applied for other mucosal bacterial infections [4]. As adjuvants for mucosal immunization, Cholera Toxin (CT) and the heat-labile toxin of enterotoxigenic *Escherichia coli* (LT) have been the most widely used in mice, although they are known to have side-effects in humans, such as the development of diarrhoea, even at low doses [6,7,8,9,10,11,12,13]. Several other adjuvants have also been used in *H*. *pylori* vaccination studies. These include linear polysaccharides such as chitosan [14] and immunostimulatory CpG oligonucleotides [15,16]. Different vaccination protocols against *H*. *pylori* have already been tested in different animal models. They usually resulted in a reduction in the number of bacteria colonizing the stomach but few strategies conferred protection in terms of sterilizing immunity [17].

In a previous *H*. *suis* vaccination study in mice, prophylactic intranasal immunization with CT adjuvanted *H*. *suis* whole-cell lysate resulted in a minority of animals being *H*. *suis* negative, as shown by conventional PCR [18]. However, increased mortality rates were observed in these vaccinated and challenged animals. This side-effect has not been thoroughly investigated yet. In addition to increased mortality rates, intranasal vaccination with a CT adjuvanted subunit vaccine consisting of a combination of different *H*. *suis* proteins including the *H*. *suis* ureB and GGT, induced post-vaccination gastritis as another major side-effect. This has also been described in *H*. *pylori* vaccination studies and its role in protection remains largely unclear [19]. Besides CT adjuvanted vaccines, a saponin-based adjuvanted *H*. *suis* whole-cell lysate has been tested in mice. This vaccine formulation was administered subcutaneously and although it induced less severe adverse effects, its protective efficacy was shown to be inferior to CT based vaccine formulations.

Recent studies describe the adjuvant activity of small molecule CC chemokine receptor 4 (CCR4) antagonists [20,21]. CCR4 is expressed on regulatory T-cells (Tregs) and Th2 cells and regulates the migration of these T cell subsets in response to MDC (macrophage derived chemokine, CCL22) and TARC (thymus and activation-related chemokine, CCL17) [22,23]. CD4^+^ Tregs express high levels of CD25 (IL-2Rα) and actively control or suppress the function of both innate and adaptive immune cells [17]. One of the most important cytokines secreted by these Tregs is the anti-inflammatory interleukin-10 (IL-10) [24]. Therefore, IL-10-producing Tregs play a role in suppressing inflammation-related pathological changes. This mechanism is, however, most likely also involved in persistence of *H*. *suis* infection in its hosts due to suppression of immune responses [18,19].

**Fig 1:**
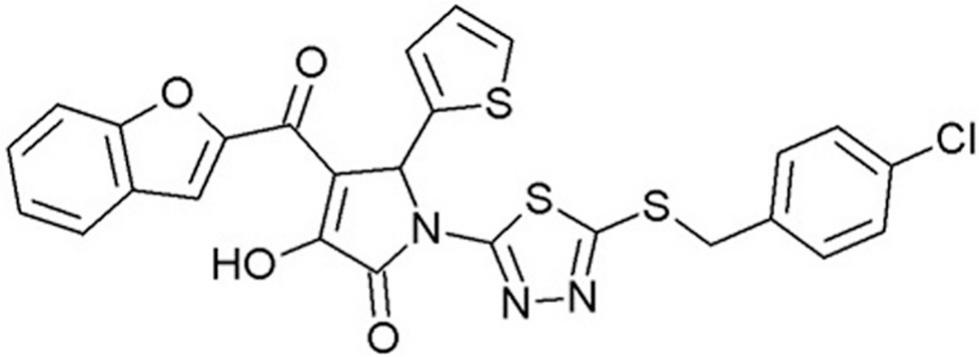
The chemical structure of the CCR4 antagonist. The CCR4 antagonist AF-399/42018025 is a small chemical molecule with a molecular weight of 565.93. It contains containing six 5 or 6 membered aromatic rings and 3 nitrogen, sulfur, and oxygen atoms. The chemical name of the molecule is 4-(1-benzofuran-2-ylcarbonyl)-1-{5-[4-chlorobenzyl)sulfanyl]-1,3,4-thiadiazol-2-yl}-3-hydroxy-5-(2-thienyl)-1,5-dihydro-2H-pyrrol-2-one [5].

CCR4 antagonists have been described to amplify cellular and humoral immune responses *in vivo* in experimental models when injected in combination with *Mycobacterium* or *Plasmodium yoelii* vaccine antigens [5,21]. In addition, CCR4 antagonists induced antigen-specific CD8^+^ T-cells and tumor immunity against self-antigens [25]. Thus far, this promising adjuvant has not been tested in vaccination and challenge studies involving pathogens. The CCR4 antagonist AF-399/42018025 used in this study is a small chemical molecule with a molecular weight of 565.93. It contains six 5 or 6 membered aromatic rings and 3 nitrogen, sulfur, and oxygen atoms. The chemical name of the molecule is 4-(1-benzofuran-2-ylcarbonyl)-1-{5-[4-chlorobenzyl)sulfanyl]-1,3,4-thiadiazol-2-yl}-3-hydroxy-5-(2-thienyl)-1,5-dihydro-2H-pyrrol-2-one [5]. (Fig 1. The chemical structure of the CCR4 antagonist).

The purpose of the first study was (1) to explore the efficacy of several established vaccine adjuvants (CpG-DNA, Curdlan, Freund’s complete (FC), Freund’s incomplete (FIC) and CT) administered subcutaneously or intranasally along with *H*. *suis* lysate to protect against *H*. *suis* challenge in mice and (2) to assess the adverse effects associated with these vaccination protocols. Since substantial side effects were observed in all vaccination strategies, a second study was performed, aiming at reducing these unwanted side effects. Administered vaccine volumes as well as routes of administration were modified and a new adjuvant, a CCR4-antagonist, was included.

## Materials and Methods

### Animals

In a first study, a total of 96 seven-week-old female *Helicobacter* specific pathogen-free BALB/c mice were purchased from an authorized breeder (HARLAN, Horst, The Netherlands). The animals were housed on autoclaved wood shavings in filter top cages. They were fed an autoclaved commercial diet (TEKLAD 2018 S, HARLAN). In a second study, a total of 66 seven-week-old female *Helicobacter* spp-free BALB/c mice were used. All animal experiments were approved by the Ethics Committee of the Faculty of Veterinary Medicine, Ghent University (EC2010/043 and EC2013/024). The animals were monitored several times a day during the whole experiment. When the animals were visibly less active, lost weight or showed symptoms of illness, the weight of the animal was compared with the other animals of the group. When a decrease of 20% of the body weight was observed, the animal was euthanized.

### Antigens for immunization

*H*. *suis* strain HS5bLP, isolated from the gastric mucosa of a sow [26], was grown on Brucella agar (Oxoid, Basingstoke, UK) supplemented with 20% fetal calf serum, 5 mg amphotericin B/l (Fungizone; Bristol-Myers Squibb, Epernon, France), *Campylobacter* selective supplement (Skirrow, Oxoid; containing 10 mg/l vancomycin, 5 mg/l trimethoprim lactate and 2500 U/l polymyxin B) and Vitox supplement (Oxoid). In addition, the pH of the agar was adjusted to 5 by adding HCl to a final concentration of approximately 0.05%. Brucella broth (Oxoid) with a pH of 5 was added on top of the agar to obtain biphasic culture conditions. After 3 days of incubation at 37°C under microaerobic conditions (85% N_2_, 10% CO_2_, 5% O_2_), the Brucella broth, containing the bacteria, was harvested [18]. Bacteria were washed, concentrated by centrifugation (5000g, 10 min, 4°C) and suspended in phosphate buffered saline (PBS). The bacterial suspension was sonicated 8 times for 30 seconds, with a frequency of 20 kHz (Sonicator ultrasonic processor XL 2015; MISONIX, Farmingdale, USA), resulting in lysis of the bacteria [18]. After centrifugation (13,000g, 10 min, 4°C), the supernatant fluid was collected and stored at -70°C until further use.

### Preparation of the challenge material

For the challenge of the mice, *H*. *suis* strain HS5bLP was cultured as described above. The final concentration was determined by counting the bacteria in an improved Neubauer counting chamber.

### Immunization/challenge study design

In the first study, the mice were divided into 10 groups (Table 1) and in the second study, 11 groups of mice were used (Table 2). In both studies, immunizations were performed under light isoflurane anaesthesia (IsoFlo; Abbott, Il, USA) on days 7, 14 and 35 after arrival. Appropriate controls were included consisting of sham-immunized mice that were either challenged (positive controls) or not (negative controls) (see Tables 1 and 2). For sham-immunization, Hank’s Balanced Salt Solution (HBSS; Invitrogen, Paisley, England) was used.

**Table 1:** Experimental set-up Study 1.

	Immunization^1^	Administration route^2^	Adjuvant/mouse^3^	Total volume administered^4^	Challenged^5^	Number of animals^6^
**1**	**Neg. Control**	IN	/	15 μl	No	6
** **	** **	SC	/	100 μl	No	6
**2**	**FC**	SC	50 μl	100 μl	Yes	12
**3**	**FIC**	SC	50 μl	100 μl	Yes	12
**4**	**Curdlan**	SC	200 μg	100 μl	Yes	12
**5**	**CT**	IN	5 μg	15 μl	Yes	12
**6**	**CpG-DNA**	IN	20 μg	15 μl	Yes	12
**7**	**Curdlan**	IN	200 μg	15 μl	Yes	12
**8**	**Pos. Control**	IN	/	15 μl	Yes	6
		SC	/	100 μl	Yes	6

Shown is the

^1^immunization protocol

^2^the administration route of the vaccine

^3^the amount of adjuvant used per mouse

^4^the volume of the vaccine

^5^the amount of lysate as shown by the protein concentration used per mouse

^6^whether the animals were challenged with *H*. *suis* or not and ^6^the number of animals in each group. (FC: Freund’s complete, FIC: Freund’s incomplete, CT: Cholera toxin, IN: intranasally, SC: subcutaneously, Pos. control.: sham-immunized/challenged, Neg. control: sham-immunized/not challenged)

**Table 2:** Experimental set up Study 2.

	Immunization^1^	Administration route^2^	Adjuvant/mouse^3^	Total volume administered^4^	Challenged^5^	Number of animals^6^
**1**	**Neg. Control**	IN	/	7 μl	No	6
**2**	**HBSS/CCR4**	SC	1.5 μg	100 μl	Yes	6
**3**	**HBSS/CCR4**	IN	1.5 μg	7 μl	Yes	6
**4**	**HBSS/CCR4**	SL	1.5 μg	7 μl	Yes	6
**5**	**CT/lysate**	IN	5 μg	7 μl	Yes	6
**6**	**CT/lysate**	SL	5 μg	7 μl	Yes	6
**7**	**FC/lysate**	SC	50 μl	100 μl	Yes	6
**8**	**CCR4/lysate**	IN	1.5 μg	7 μl	Yes	6
**9**	**CCR4/lysate**	SL	1.5 μg	7 μl	Yes	6
**10**	**CCR4/lysate**	SC	1.5 μg	100 μl	Yes	6
**11**	**Pos. Control**	IN	/	7 μl	Yes	6

Shown is the

^1^immunization protocol

^2^the administration route of the vaccine

^3^the amount of adjuvant used per mouse

^4^the volume of the vaccine

^5^the amount of lysate as shown by the protein concentration used per mouse

^6^whether the animals were challenged with *H*. *suis* or not and ^6^the number of animals in each group. (HBSS: Hank’s balanced salt solution, CCR4: CC chemokine receptor 4 antagonist, CT: Cholera toxin, FC: Freund’s complete, IN: intranasally, SC: subcutaneously, SL: sublingually, Neg. control: sham-immunized/not challenged, Pos. control: sham-immunized/challenged,)

Several adjuvants were used, inducing different immune responses. Curdlan is known to elicit a predominant Th17 response [27]. CpG-DNA and Freund’s complete are known to elicit a predominant Th1 response, whereas Freund’s incomplete and Cholera toxin elicit a predominant Th2 response [28]. In the first study, subcutaneous vaccination was done by mixing 100 μg of *H*. *suis* sonicate (in a volume of 50 μl) with an equal volume of FC (Sigma-Aldrich, St. Louis, MO, USA), FIC (Sigma-Aldrich, St. Louis, MO, USA) or 200 μg Curdlan (Sigma-Aldrich, St. Louis, MO, USA). This mixture was injected at the lower back of the animals. For intranasal vaccination, a total volume of 15 μl was used, containing 100 μg of *H*. *suis* sonicate mixed with 5 μg CT (Sigma-Aldrich, St. Louis, MO, USA), 20 μg CpG-DNA (Hycult, biotech, The Netherlands) or 200 μg Curdlan (Sigma-Aldrich, St. Louis, MO, USA). The mixture was then applied on the external nares of the mice.

In the second study, subcutaneous vaccination was done by mixing 100 μg of *H*. *suis* sonicate with 1.5 μg CCR4 antagonist or 50 μl FC. For sublingual and intranasal vaccination, a total volume of 7 μl was used, containing 100 μg of *H*. *suis* sonicate mixed with 5 μg CT or 1.5 μg CCR4 antagonist.

On day 56 of both studies, the mice were intragastrically inoculated with 0.3 ml of the challenge material, containing approximately 2 x 10^8^ viable *H*. *suis* bacteria/ml, using a ball-tipped gavage needle. The mice were held in an upright position until they regained consciousness, to minimize the risk of reflux.

In both studies, all animals were euthanized on day 77 by cervical dislocation under deep isoflurane anaesthesia (IsoFlo; Abbott, Il, USA). The stomachs were removed and preserved in RNAlater (Ambion, Austin, TX, USA) at -70°C until DNA and RNA extraction. A longitudinal strip of tissue taken along the curvatura major of the stomach was fixed in phosphate buffered formaline (4%) for histopathological analyses. For DNA and RNA isolation, the stomachs were homogenized in 1 ml Tri-reagent (MRC, Brunschwig Chemie, Amsterdam, The Netherlands) using a MagnaLyser (Roche Applied Science, Mannheim, Germany). The interphase and organic phase were kept at -20°C for subsequent DNA extraction according to the instructions of the manufacturer. Meanwhile, total RNA was extracted from the upper aqueous phase using the RNeasy mini kit (Qiagen, Venlo, The Netherlands).

### Quantitative Real-Time PCR for the quantification of the bacteria

Quantitative Real-Time PCR (RT-PCR) was performed using the C1000 Thermal cycler (CFX96 Real-Time System, Bio-Rad, Hercules, CA, USA). Each sample contained 5 μl of iQ SYBR Green Supermix (Bio-Rad), 0.25 μl of each primer, 3.5 μl of distilled water and 1 μl of DNA.

For enumeration of colonizing *H*. *suis* bacteria in the first study, a 218 bp fragment of the *ureB* gene of *H*. *suis* was amplified using the following primers: sense primer: 5’-TTA CCA AAA ACA CCG AAG CC-3’, antisense primer: 5’-CCA AGT GCG GGT AAT CAC TT-3’. A 1146 bp PCR fragment of the *ureB* gene served as an external standard (sense primer: 5’- CGG GAT TGA TAC CCA CAT TC-3’; antisense primer: 5’- ATG CCG TTT TCA TAA GCC AC-3’) [29].

For enumeration of colonizing bacteria in the second study, a fragment of the UreA gene of *H*. *suis* was amplified using the following primers: sense primer BF_HsuisF1: 5′-AAA ACA MAG GCG ATC GCC CTG TA-3′ and anti-sense primer BF_HsuisR1: 5′-TTT CTT CGC CAG GTT CAA AGC G-3′. For generating the standard, part of the *ureAB* gene cluster (1236 bp) from *H*. *suis* strain HS5 was amplified using primers U430F and U1735R, as described previously [30].

The copy number concentration was calculated based on the length of the amplicon and the mass concentration. The standard consisted of 10-fold dilutions starting at 10^7^ PCR amplicons for each 10 μl of reaction mixture. Data analysis was done using the Bio-Rad CFX Manager Version 3.0 software (Bio-Rad).

### Quantitative Real-Time PCR for the analysis of cytokine and chemokine expression

Messenger RNA expression levels in stomach tissue were determined for the following genes: IL-17, IL-4, IFN-γ, keratinocyte chemoattractant (KC or CXCL1), LPS-induced CXC chemokine (LIX), macrophage inflammatory protein-2 (MIP-2 or CXCL2) and IL-10. For the RT-PCR reaction, a C1000 Thermal cycler (CFX96 Real-Time System, Bio-RAD, Hercules, CA, USA) was used. All reactions were performed in a final volume of 10 μl containing 5 pmole of the sense and 5 pmole of the antisense primers, 5 μl iQ SYBR mix (Bio-Rad) and 1 μl cDNA. The reaction protocol consisted of an initial activation phase at 95°C for 15 minutes followed by 40 cycles of 95°C for 20 seconds, 60°C for 30 seconds and 73°C for 30 seconds. A melting curve was included by increasing the temperature with 0.5°C every 5 seconds starting from 65°C until 95°C. The housekeeping genes H2afz, PPIA and HPRT were shown to have a stable expression in all samples tested (data not shown) and were included as references. The sequences of all used primers are summarized in Table 3.

**Table 3:** List of genes and sequences of the primers used for RT-PCR gene expression analysis [28].

Gene	Primer	Primer Sequence
IFN-γ	sense	5'-GCG TCA TTG AAT CAC ACC TG-3'
	antisense	5'-TGA GCT CAT TGA ATG CTT GG-3'
IL-4	sense	5'-ACT CTT TCG GGC TTT TCG AT-3'
	antisense	5'-AAA AAT TCA TAA GTT AAA GCA TGG TG-3'
IL-10	sense	5'-ATC GAT TTC TCC CCT GTG AA-3'
	antisense	5'-CAC ACT GCA GGT GTT TTA GCT CC-3'
IL-17	sense	5'-TTT AAC TCC CTT GGC GCA AAA-3'
	antisense	5'-CTT TCC CTC CGC ATT GAC AC-3'
KC	sense	5'-GCT GGG ATT CAC CTC AAG AA-3'
	antisense	5'-TCT CCG TTA CTT GGG GAC AC-3'
MIP-2	sense	5'-AAA GTT TGC CTT GAC CCT GA-3'
	antisense	5'-TCC AGG TCA GTT AGC CTT GC-3'
LIX	sense	5'-CCC TGC AGG TCC ACA GTG CC-3'
	antisense	5'-TGG CCG TTC TTT CCA CTG CGA-3'
H2afz	sense	5’-CGT ATC ACC CCT CGT CAC TT-3’
	antisense	5’-TCA GCG ATT TGT GGA TGT GT-3’
PPIA	sense	5’-AGC ATA CAG GTC CTG GCA TC-3’
	antisense	5’-TTC ACC TTC CCA AAG ACC AC-3’
HPRT	sense	5’-CAG GCC AGA CTT TGT TGG AT-3’
	antisense	5’-TTG CGC TCA TCT TAG GCT TT-3’

The threshold cycle values (Ct) were first normalized to the geometric means of the reference genes and the normalized mRNA levels were calculated according to 2^(-∆∆Ct)^ method for each individual animal [31].

### Histopathological evaluation of the gastric wall

Samples of the gastric wall were formalin-fixed, paraffin wax embedded, cut at 5 μm and stained with hematoxylin and eosin according to standardized protocols. Inflammation (infiltration of granulocytes as well as mononuclear cells) was scored separately for the mucosa and submucosa, using a scoring system adapted from the human updated Sydney System [32]. Scoring of inflammatory cell infiltration was performed as follows: 0 = normal, 1 = multifocal mild, 2 = diffuse mild or multifocal moderate, 3 = diffuse mild and (multi)focal moderate, 4 = diffuse moderate or multifocal severe, 5 = diffuse moderate and (multi)focal severe, 6 = diffuse severe. Numbers of globular leukocytes were scored as follows: 0 = no globular leukocytes, 1 = mild increase in globular leukocyte numbers, 2 = moderate increase in globular leukocyte numbers, 3 = severe increase in globular leukocyte numbers. In the second study, the presence of lymphocyte and neutrophil infiltration in the mucosa of the fundus was scored separately as follows: 0 = normal, 1 = mild, 2 = moderate, 3 = severe. In addition, Periodic acid-Schiff (PAS) staining was performed to evaluate the presence of pseudo pyloric metaplasia of the fundus.

### Statistical analysis

For all analyses, SPSS 22 (IBM, USA) was used. Differences in *H*. *suis* colonization and cytokine expression among groups were assessed by using one-way ANOVA analysis. A Bonferroni post-hoc test was used for comparisons between the different groups. Histological inflammation scores were compared by a Kruskal-Wallis analysis, followed by a Mann-Whitney U test. For correlations between different variables, Spearman’s rho coefficient (ρ) was calculated. A level of probability of 0.05 was used as criterion for significance.

## Results

### Cholera toxin, Freunds’ complete adjuvant and CCR4 antagonists confer the highest level of protection against colonizing *H*. *suis* bacteria

**Fig 2:**
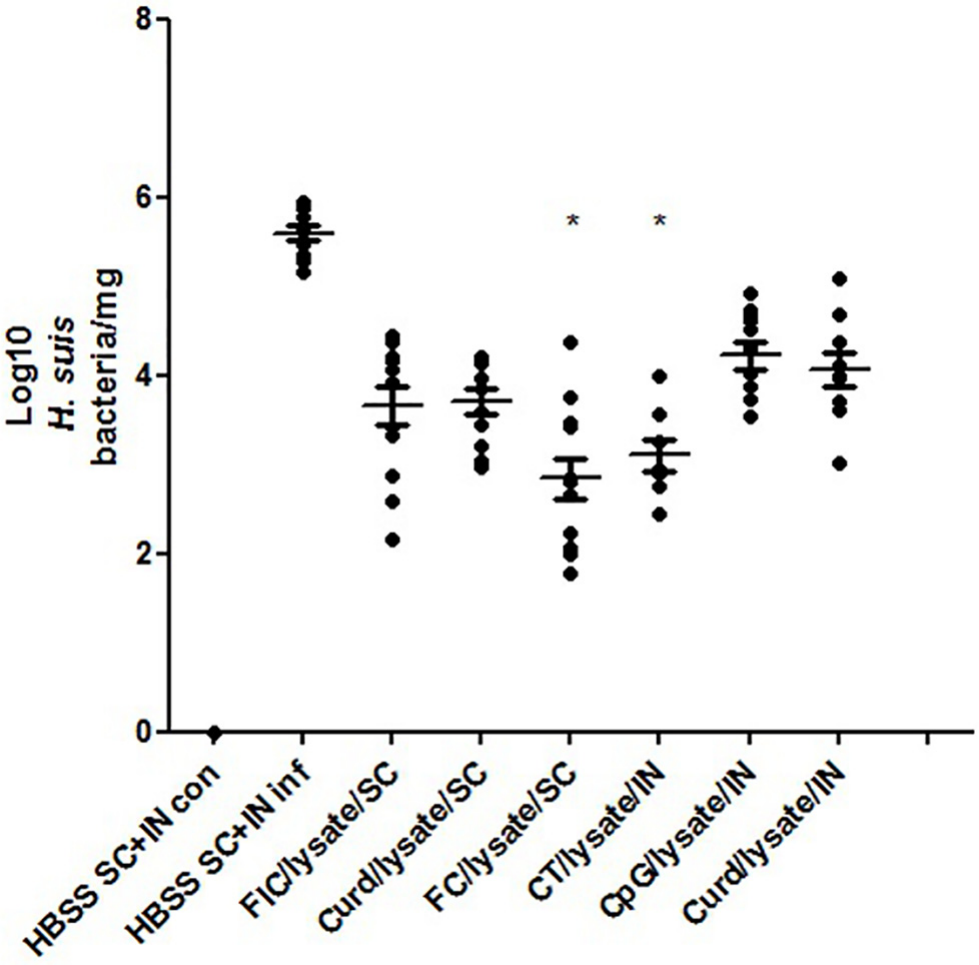
The protective efficacy of different adjuvants on the amount of colonizing *H*. *suis* bacteria, 3 weeks after challenge, in study 1. Subcutaneous immunization (SC) was done by mixing *H*. *suis* sonicate with Freund’s complete (FC), Freund’s incomplete (FIC) or Curdlan (Curd) and injecting this mixture at the lower back of the mice. Intranasal immunization (IN) was done by mixing *H*. *suis* sonicate with Cholera toxin (CT), CpG-DNA (CpG) or Curdlan (Curd) and applying this mixture on the external nares of the mice. Animals in the control groups were sham-immunized with Hank’s Balanced Salt Solution (HBSS). The bacterial load is illustrated as log(10) of *H*. *suis* copies/mg stomach tissue. Individual mice are illustrated as dots. Significant differences between immunized and non-immunized challenged animals are noted by * (p<0.05). (con: uninfected, inf: infected).

In the first study, all immunization strategies with various adjuvants and routes of immunization (i.e. subcutaneous immunization with FC, FIC, Curdlan or intranasal immunization with CT, CpG and Curdlan) resulted in the reduction of bacterial burden following *H*. *suis* challenge, when compared to the sham-immunized/challenged animals (Fig 2). However, striking differences were observed among various adjuvants in their capacity to decrease the amount of colonizing *H*. *suis* bacteria. Subcutaneous FC/lysate (p<0.05) administration and intranasal CT/lysate (p<0.001) administration were the only to induce significant protection. (Fig 2. The protective efficacy of different adjuvants on the amount of colonizing *H*. *suis* bacteria, 3 weeks after challenge in study 1)

**Fig 3:**
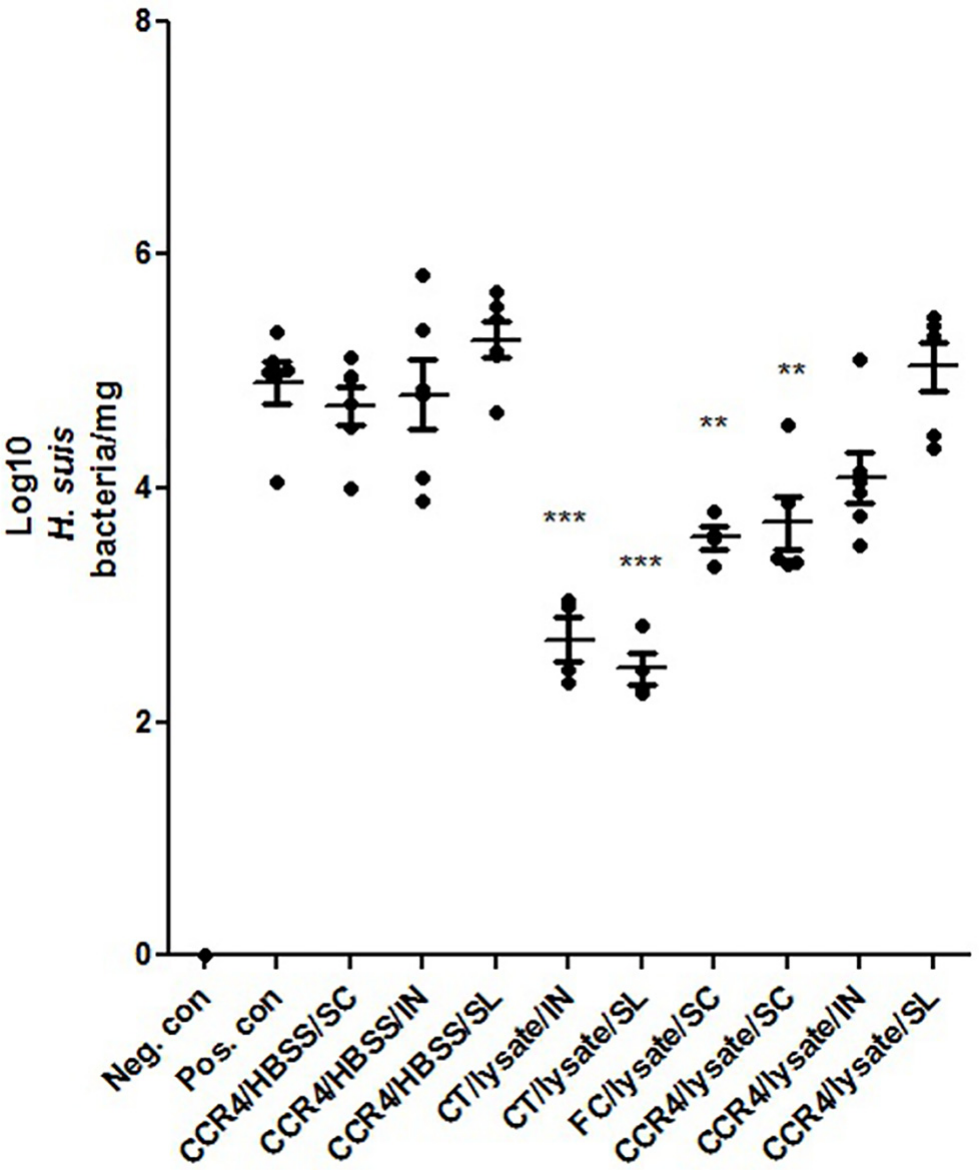
The protective efficacy of the different immunization protocols on the amount of colonizing *H*. *suis* bacteria, 3 weeks after challenge, in study 2. Subcutaneous immunization (SC) was done by mixing *H*. *suis* sonicate with Freund’s complete (FC) or the CCR4 antagonist (CCR4) and injecting this mixture at the lower back of the mice. Intranasal immunization (IN) was done by mixing *H*. *suis* sonicate with Cholera toxin (CT) or the CCR4 antagonist (CCR4) and applying this mixture on the external nares of the mice. Sublingual immunizations (SL) were done by mixing *H*. *suis* sonicate with Cholera toxin (CT) or the CCR4-antagonist (CCR4). Animals in the control groups were sham-immunized with Hank’s Balanced Salt Solution (HBSS). The amount of bacteria colonizing the stomach of the mice are illustrated as log(10) of *H*. *suis* copies/mg stomach. The individual animals are presented as dots. Significant differences between the immunized and challenged groups and the positive control group are noted by ** (p<0.01) and *** (p<0.001). (neg. con.: sham-immunized/not challenged, pos. con.: sham-immunized/challenged).

The results obtained with CT/lysate were also confirmed in the second study, wherein the intranasal as well as sublingual route of immunization conferred the highest protection against colonization of *H*. *suis* in the stomach (P<0.001) (Fig 3). In addition, animals subcutaneously immunized with FC/lysate and CCR4 antagonist/lysate showed significantly lower *H*. *suis* colonization levels after experimental challenge (P<0.01). In contrast, mucosal immunization with CCR4 antagonists (both intranasally and sublingually) and lysate showed no significant decrease in bacteria colonizing the stomach. Colonization levels in animals that were administrated the CCR4 antagonist in the absence of lysate and that were subsequently challenged, were similar to those in the sham-immunized and challenged positive control animals. (Fig 3. The protective efficacy of the different immunization protocols on the amount of colonizing *H*. *suis* bacteria, 3 weeks after challenge in study 2)

### Mice immunized with CCR4 antagonists display minimal pseudo-pyloric metaplasia as compared to the Freund’s complete adjuvant group upon challenge with *H*. *suis*

In both studies, a number of animals died in each group immunized with CT (both intranasally and sublingually), within 48 hours after challenge with *H*. *suis*. The most likely cause was an immunization/challenge related pneumonia, for histopathological evaluation showed the presence of oedema in the interstitial space of the lungs of these mice. In the first study, two mice from the Curdlan/intranasally vaccinated group died before challenge, and one mouse from the CpG/intranasally vaccinated group died of an unknown cause at week eleven following challenge.

**Fig 4:**
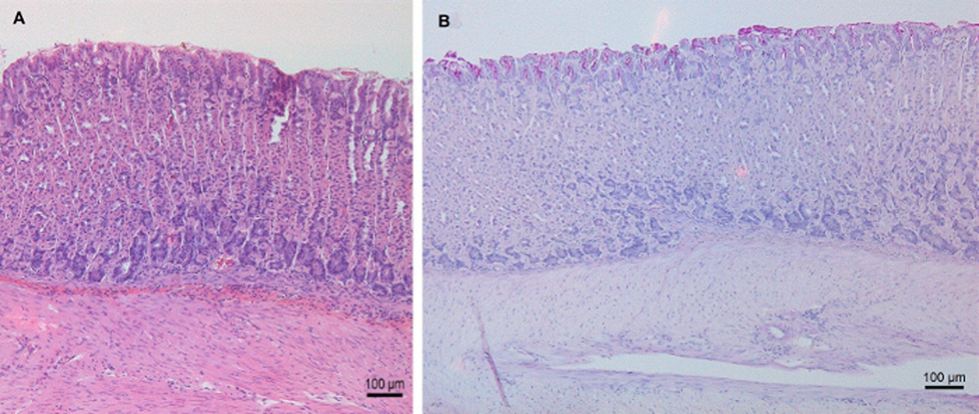
H&E and PAS staining of a normal fundus. H&E (A) and PAS staining (B) of a normal fundus of an animal from the negative control group (original magnification: 100x).

In both studies, all negative control mice showed normal histomorphology with very little to no inflammatory cell infiltration in the gastric mucosa (Fig 4). All positive control groups showed moderate to severe inflammation. (Fig 4. H&E and PAS staining of a normal fundus)

In the first study, mice immunized subcutaneously with FIC/lysate, FC/lysate and Curdlan/lysate clearly showed a stronger infiltration of granulocytes in the fundic mucosa and submucosa (p<0.05) (Table 4) as compared to animals from the intranasally immunized/challenged groups. Detection of globular leukocytes was restricted to the mucosa of the subcutaneously immunized groups. In addition, all immunized and challenged groups showed moderate lymphocyte infiltration, and this was most pronounced in the group immunized with CT/lysate (Table 4).

**Table 4:** Histopathological scoring of inflammation Study 1.

	Pseudo-pyloric metaplasia	Granulocyte infiltration	Mononuclear cell infiltration
**HBSS/sc/con**	0 (0–0)	0 (0–1)	0 (0–0.5)
**HBSS/in/con**	0 (0–0)	0 (0–0)	0 (0–0.5)
**HBSS/sc/inf**	0 (0–0)	1 (0.5–1)	0.5 (0.5–1.5)
**HBSS/in/inf**	0 (0–0)	1 (0.5–2.5)	1.5 (0.5–1.5)
**FC/lysate/SC**	2.5 (0–3)*	1.5 (0.5–2)*	0.5 (0.5–1)
**FIC/lysate/SC**	3 (3–3)*	1.5 (0,5–2)*	0.5 (0–0.5)
**Curd/lysate/SC**	3 (3–3)*	1.5 (0.5–2)*	0.5 (0.5–1)
**CT/lysate/IN**	0 (0–0)	0.5 (0–0.5)	0.5 (0.5–1)
**CpG/lysate/IN**	0 (0–0)	0 (0–1)	0.5 (0.5–1)
**Curd/lysate/IN**	0 (0–0)	0 (0–0.5)	0.5 (0.5–0.5)

Shown are the median scores (min-max) of pseudo-pyloric metaplasia of the fundus, infiltration of the mucosa of the fundus with granulocytes and mononuclear cells 3 weeks after challenge of the vaccinated mice. Scoring: 0 = normal, 1 = mild, 2 = moderate, 3 = severe. Significant differences between the positive control group and the immunized and challenged groups are noted by

* (p<0.05). (HBSS: Hank’s balanced salt solution FC: Freund’s complete, FIC: Freund’s incomplete, CT: Cholera toxin, Curd: Curdlan, CpG: CpG-DNA IN: intranasally, SC: subcutaneously, con: uninfected group, inf: infected group)

Results from the second study revealed that, compared to the positive control group, mice that received CT/lysate by the sublingual route showed significantly increased neutrophil infiltration in the fundus (p<0.01) upon challenge with *H*. *suis*. In addition, compared to the positive control groups, lymphocyte infiltration was significantly increased in all the immunized and challenged groups except for the groups immunized with the CCR4 antagonist/lysate by sublingual and intranasal routes (Table 5).

**Table 5:** Histopathological scoring of inflammation Study 2.

	Pseudo-pyloric metaplasia	Granulocyte infiltration	Mononuclear cell infiltration
**Neg. Control**	0 (0–0)	0 (0–0)	0 (0–0)
**CCR4/SC**	0 (0–0)	1.5 (0–2)	1.5 (0–2)
**CCR4/IN**	0 (0–0)	0.5 (0–2)	0.5 (0–1)*
**CCR4/SL**	0 (0–0)	1 (0–2)	1.5 (1–2)*
**CT/lysate/IN**	0 (0–0)	1.5 (0–3)	2 (1–3)
**CT/lysate/SL**	1.5 (1–2)*	2.5 (2–3)*	3 (3–3)*
**FC/lysate/SC**	3 (3–3)*	1.5 (1–2)	2.5 (2–3)
**CCR4/lysate/IN**	0 (0–0)	1 (0–1)*	0.5 (0–1)*
**CCR4/lysate/SL**	0.5 (0–1)	0.5 (0–1)*	0.5 (0–1)*
**CCR4/lysate/SC**	2 (0–3)*	1 (1–2)	2.5 (1–3)
**Pos. Control**	1.5 (0–2)	1 (1–1)	2 (1–3)

Shown are the median scores (min-max) of pseudo-pyloric metaplasia of the fundus, infiltration of the mucosa of the fundus with granulocytes and mononuclear cells 3 weeks after challenge of the vaccinated mice. Scoring: 0 = normal, 1 = mild, 2 = moderate, 3 = severe. Significant differences between the positive control group and the immunized and challenged groups are noted by * (p<0.05). (CCR4: CC chemokine receptor 4 antagonist, CT: Cholera toxin, FC: Freund’s complete, IN: intranasally, SC: subcutaneously, SL: sublingually, Neg. control: sham-immunized/not challenged, Pos. control: sham-immunized/challenged)

**Fig 5:**
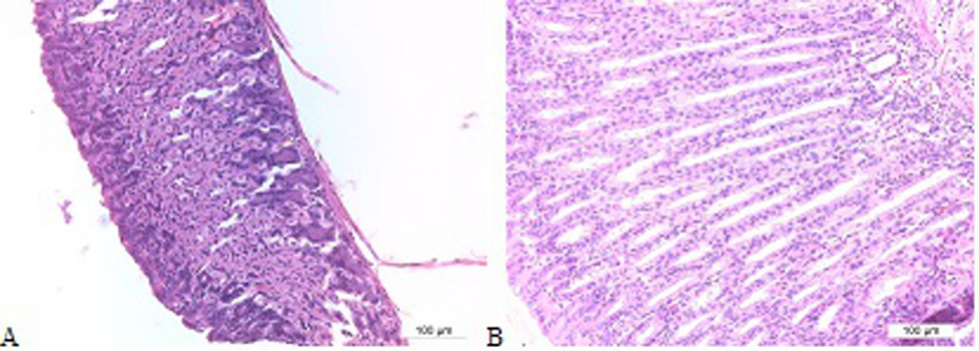
Histopathological analysis of fundus by H&E staining. Normal fundus from an uninfected BALB/c mouse (A). Metaplastic fundus of a Freund’s complete/lysate immunized and *H*. *suis*-infected BALB/c mouse, showing severe parietal cell loss, with replacement of the intestinal epithelium by a common glandular epithelium (B). Original magnification: 200x.

**Fig 6:**
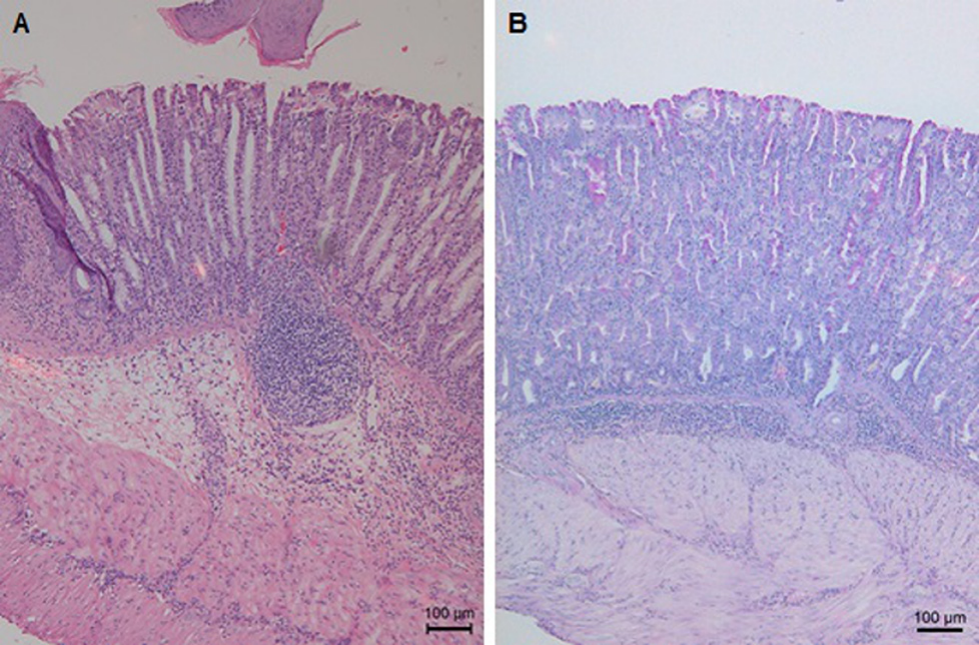
H&E and PAS staining of the fundus showing pronounced inflammation and severe pseudo-pyloric metaplasia. H&E (A) and PAS staining (B) of the fundus of an animal in the FC subcutaneously immunized and challenged group, showing pronounced inflammation and severe pseudo-pyloric metaplasia (original magnification: 100x).

**Fig 7:**
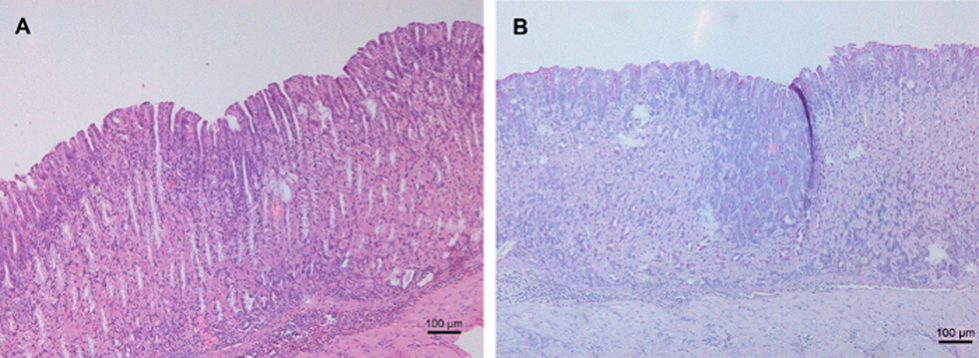
H&E and PAS staining of the fundus, showing mild inflammation and pseudo-pyloric metaplasia. H&E (A) and PAS staining (B) of the fundus of an animal from the CCR4 antagonist subcutaneously immunized and challenged group, showing mild inflammation and pseudo-pyloric metaplasia (original magnification: 100x).

Interestingly, in the first study, pseudo-pyloric metaplasia was detected in the stomach of 75% of the mice from the FC/lysate immunized and challenged group and even in 100% of the animals belonging to the FIC/lysate and SC Curdlan/lysate immunized and challenged animals. In all other groups, no pseudo-pyloric metaplasia was observed (Fig 5). In the second study, pseudo-pyloric metaplasia of the fundus was present in the group immunized with FC/lysate and CT/lysate/SL and challenged (Fig 6). Although pseudo-pyloric metaplasia was also present in the CCR4 antagonist/lysate/SC immunized and challenged group (Fig 7), this was nevertheless less severe and not present in all animals, compared to the group immunized with FC/lysate (Table 5). (Fig 5. Histopathological analysis of fundus by H&E staining, Fig 6. H&E and PAS staining of the fundus showing pronounced inflammation and severe pseudo-pyloric metaplasia, Fig 7. H&E and PAS staining of the fundus, showing mild inflammation and pseudo-pyloric metaplasia)

### Immunization with adjuvants modulates the cytokine responses in the stomach upon *H*. *suis* challenge

**Fig 8:**
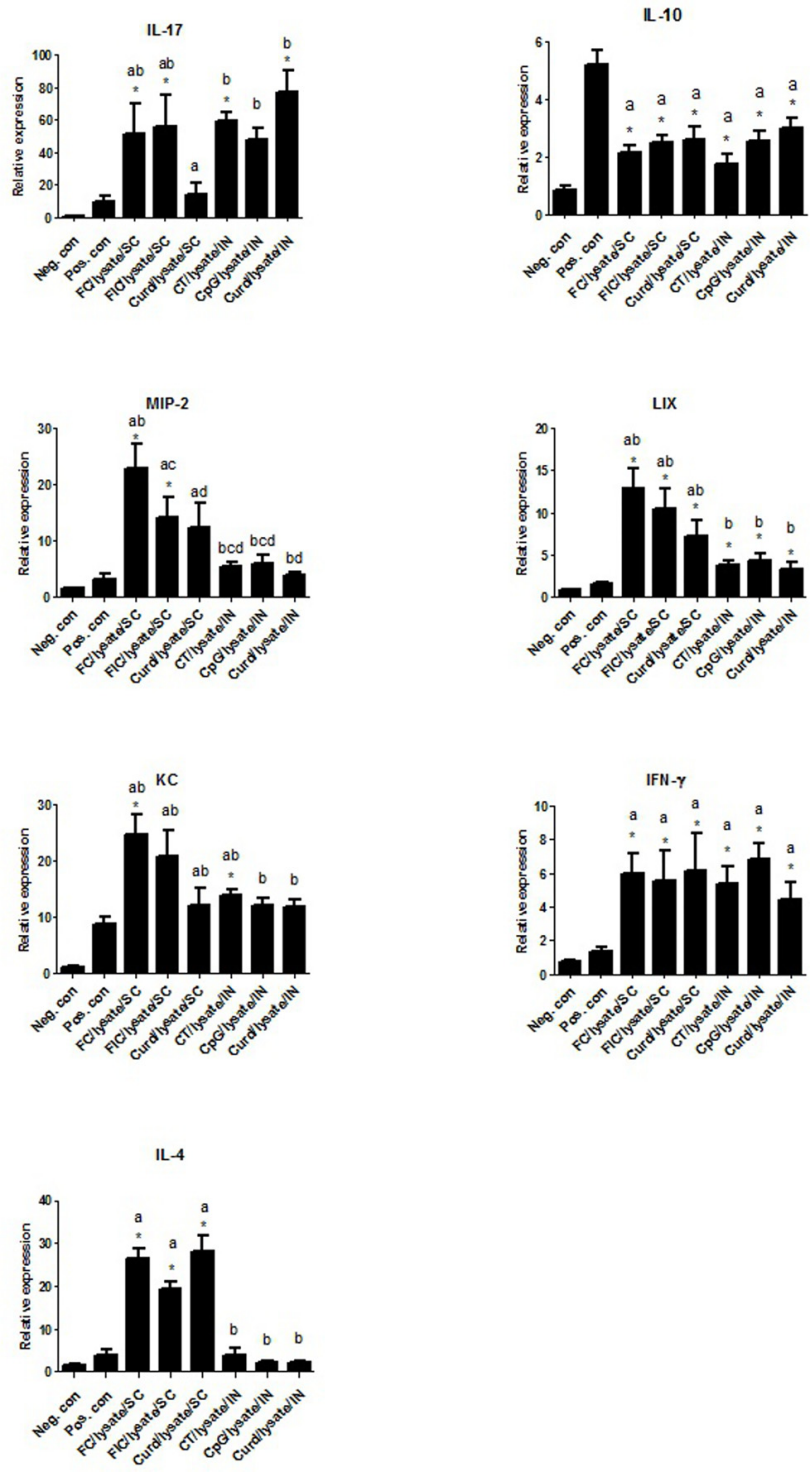
Relative gene expression of cytokines and chemokines in the stomach of challenged animals after immunization or after sham inoculation in study 1. The first bar represents the pooled data of the animals that were sham-immunized with HBSS (intranasally and subcutaneously) and that were not challenged with *H*. *suis* (Neg. con). The second bar represents the pooled data of the animals that were sham-immunized with HBSS (intranasally and subcutaneously) and that were challenged with *H*. *suis* (Pos. con). Bars 3, 4 and 5 represent the groups of animals that were immunized subcutaneously with Freund’s complete (FC/lysate/SC), Freund’s incomplete (FIC/lysate/SC) or Curdlan (Curd/lysate/SC) and challenged with *H*. *suis*. Bars 6, 7 and 8 represent the animals that were immunized intranasally with Cholera Toxin (CT/lysate/IN), CpG-DNA (CpG/lysate/IN) or Curdlan (Curd/lysate/IN) and challenged with *H*. *suis*. An * (p<0.05) indicates a significant modulation of mRNA expression levels compared to the sham-immunized/challenged groups. Cytokine expression between immunized and challenged groups was also compared with each other. When no differences could be found between the different immunized groups, the same letter designation was attributed.

In the first study, data from immunized/challenged groups were compared to pooled data from the sham-immunized/challenged positive control groups (Fig 8). Expression of IL-10, an important immunoregulatory cytokine, was significantly lower in all the adjuvant groups as compared to the sham-immunized and challenged groups (p<0.0001). In the subcutaneously immunized/challenged groups, adjuvants showed distinct differences in their ability to mount helper T cell responses. Although FC/lysate, FIC/ lysate and and Curdlan/lysate groups showed significantly higher Th1 and Th2 cytokine signatures (IFN-γ; p<0.0001 and IL-4; p<0.01), Th17 cytokine responses (IL-17) were only observed with Freund’s complete and incomplete adjuvant groups. Mice immunized by mucosal routes displayed distinct helper T cell responses as compared to subcutaneous immunization routes. No Th2 response (IL-4) was induced in CT/lysate, CpG/lysate and Curdlan/lysate immunized groups, suggesting that these adjuvants selectively induce Th1 and Th17 responses when administered by mucosal routes. Clearly, Curdlan/lysate, irrespective of the route of vaccination, was less efficient to induce chemokine expression as compared to Freund’s adjuvants. In all immunized/challenged animals, LIX showed higher mRNA expression levels (p<0.05) when compared to the sham-immunized/challenged animals. This was not the case for MIP-2 and KC. However, a clear upregulation of MIP-2 mRNA expression levels was observed in the FC/lysate and FIC/lysate groups, while a clear upregulation of KC expression was only observed in the FC/lysate and CT/lysate groups. Messenger RNA expression levels of these cytokines are presented in Fig 8. (Fig 8. Relative gene expression in the stomach in challenged animals after immunization or after sham inoculation in study 1)

**Fig 9:**
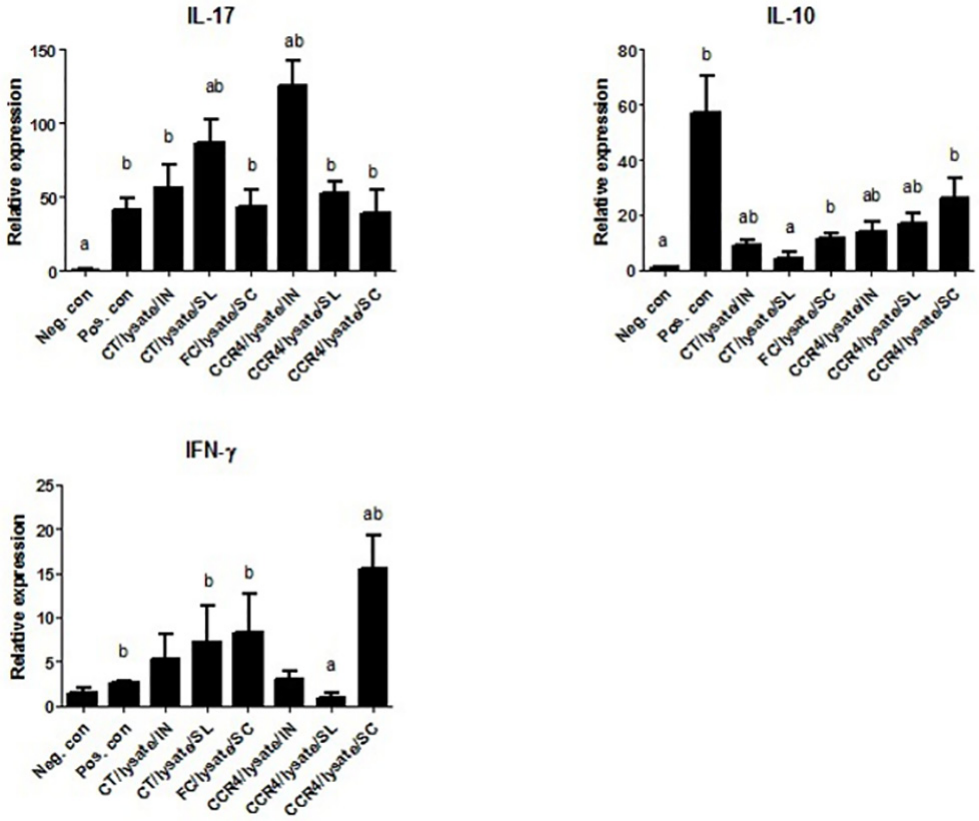
Relative gene expression in the stomach in challenged animals after immunization or after sham inoculation in study 2. The first bar represents the pooled data of negative control animals that were sham-immunized with HBSS and remained unchallenged (Neg. con). The second bar represents positive control group where mice were sham-immunized with HBSS (intranasally and subcutaneously) and were subsequently infected with *H*. *suis* (Pos. con). Bars 3 and 4 represent Cholera Toxin groups immunized intranasally (CT/lysate/IN) or sublingual routes (CT/lysate/SL) and challenged with *H*. *suis*. Bar 5 represents Freund’s complete adjuvant group (FC/lysate/SC). Bars 6 to 8 denote CCR4 antagonists group immunized by intranasal (IN), sublingual (SL) or subcutaneous (SC) routes respectively followed by challenge with *H*. *suis*. The letter ‘a’ indicates a significant (p<0.05) difference of mRNA expression levels compared to positive control groups. The letter ‘b’ indicates significant (p<0.05) changes of expression levels compared to the negative control groups.

Similar results were obtained in the second study (Fig 9). IL-10 expression was clearly lower in all vaccinated groups as compared to positive control groups. Of note, the use of the CCR4 antagonist by the subcutaneous route induced highest IFN-γ responses confirming the previous results obtained with this adjuvant in other models where cellular immune responses were significantly induced [21,25]. However, Th1 responses were not induced when the CCR4 antagonist was used for mucosal immunization, a protocol which was shown not to be able to confer protection upon challenge with *H*. *suis*. (Fig 9. Relative gene expression in the stomach in challenged animals after immunization or after sham inoculation in study 2)

**Fig 10:**
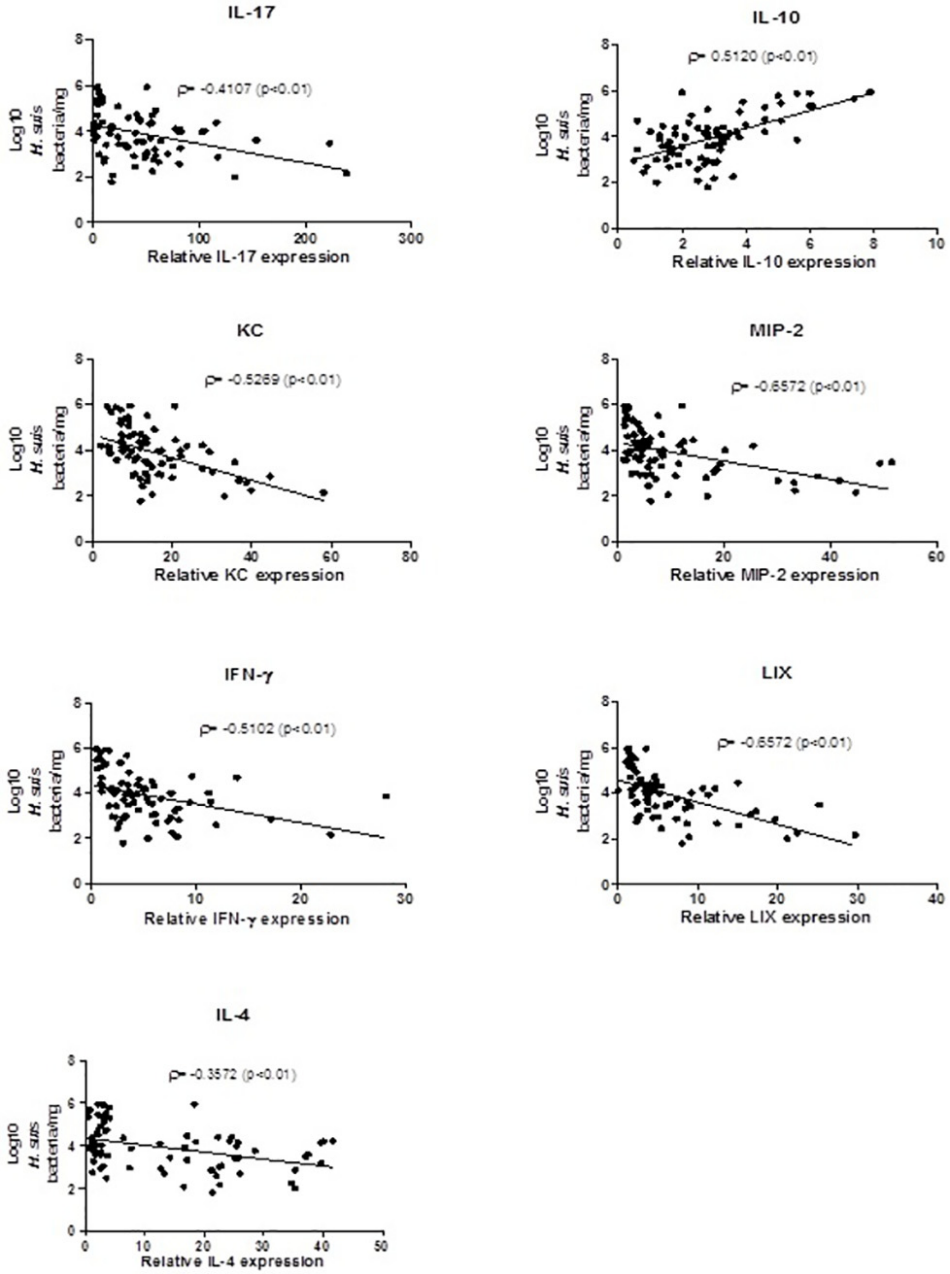
Correlation between cytokine and chemokine expression and *H*. *suis* colonization in study 1. Shown are the correlation analyses between IL-17, IL-10, MIP-2, LIX, IFN-γ and IL-4 mRNA expression levels on the one hand and the number of *H*. *suis* bacteria colonizing stomach of mice in the immunized and challenged groups on the other hand. Correlation was measured by Spearman’s Rho (ρ). Statistical significance between the immunized and challenged groups and the positive control group is noted by the P-value.

In both studies, correlation analysis showed a clear, positive correlation between IL-10 expression and *H*. *suis* colonization rates. A significant inverse correlation was found between the mRNA expression levels of helper T cell cytokines and chemokines tested and the bacterial load (Fig 10). (Fig 10. Correlation between cytokine expression and *H*. *suis* colonization in study 1)

## Discussion

In a previous study, we showed that intranasal immunization, with a CT/*H*. *suis* lysate vaccine, induced a partial protection against subsequent *H*. *suis* challenge, with some animals even showing no detectable *H*. *suis* colonization by conventional PCR [18]. In this same study, animals were also immunized subcutaneously using a *H*. *suis* lysate/saponin-based adjuvant vaccine, which clearly was shown to be inferior to the intranasal immunization protocol, with regards to its capacity to protect against a subsequent *H*. *suis* challenge [18]. Our current results confirm that the choice of adjuvant plays an important role in the efficacy of a vaccine formulation. Indeed, subcutaneous vaccination with *H*. *suis* lysate adjuvanted with FC or with the CCR4 antagonist induced a similar protection compared to intranasal vaccination with CT as an adjuvant. In contrast to parenteral immunization, mucosal immunization using this CCR4 antagonist as an adjuvant did not induce a good protection against *H*. *suis* challenge. CCR4+ effector memory regulatory T cells (Tregs) have homing capacity to skin and lungs [22]. Therefore, the use of CCR4 antagonists as molecular adjuvant is strictly dependent on the route of immunization. It is possible that lack of migration of CCR4+ Tregs to the sublingual region might have contributed to ineffectiveness of this route of vaccination with CCR4 antagonists. Conflicting results have been reported when comparing the efficacy of mucosal versus systemic immunization routes for *Helicobacter* vaccination, but most studies suggest that mucosal immunization is the better one of the two options [12,13,33]. Results from the present study however indicate that subcutaneous vaccination, using various well-established vaccine adjuvants, in general yields the best results with regards to reduction of *H*. *suis* colonization. The only mucosally applied adjuvant generating similar levels of protection was CT, which was shown to be equally effective when used for sublingual immunization (Fig 3).

In the present study, protection was obtained in mice immunized subcutaneously with FC as adjuvant, which was accompanied by high expression rates of MIP-2, LIX and KC (Fig 8). These chemokines are known to be involved in the recruitment of neutrophils. Indeed, infiltration with (neutrophilic) granulocytes was more pronounced in subcutaneously immunized mice, both compared to intranasally immunized/challenged mice as well as sham-immunized/challenged positive control animals. This however contrasts somewhat with the results of our second study, showing an increased infiltration with neutrophils in animals immunized mucosally using CT as an adjuvant. In any case, a more pronounced post-vaccination gastritis was associated with a higher degree of protection observed in both studies. This is in accordance with data found in literature for *H*. *pylori* vaccination [8,34,35]. However, the disparity in gastritis between infected animals and immunized/challenged animals has been described to disappear, showing that this is most likely a transient event [8,12]. Whether post vaccination gastritis is also a transient event in *H*. *suis* vaccination and subsequent challenge remains to be determined.

Compared to the non-immunized/challenged animals, IL-10 expression levels were consistently lower in animals from all immunized/challenged groups. In addition, a mild positive correlation between IL-10 expression and gastric *H*. *suis* colonization rates was demonstrated (Fig 10). It has been suggested that *H*. *suis* and *H*. *pylori* suppress the generation of an efficient immune response by inducing expression of anti-inflammatory/regulatory IL-10, which contributes to the suppression of pro-inflammatory Th17 and Th1 responses [18,36]. IL-10 is one of the most important cytokines secreted by regulatory T-cells (Tregs), which actively control or suppress the function of other cells. Infection of IL-10^-/-^ mice, lacking IL-10 expression, with *H*. *pylori* and *H*. *felis* elicited a more severe chronic gastritis compared with that seen in wild-type mice [37], which even led to spontaneous eradication of *Helicobacter* infection [4,38]. Kao et al. showed that mucosal dendritic cells induce a Treg-biased response suppressing the induction of a Th17 response, which could in part be responsible for the chronicity of the infection [36]. Since Tregs and IL-10 may contribute to persistence of an infection with *H*. *suis*, we hypothesized that CCR4 antagonists might help to induce protective immunity. Results of our study indeed reveal that a significant level of protection was achieved when using the CCR4 antagonist as an adjuvant and this was associated with only a mild pseudo-pyloric metaplasia, which was sometimes even absent. By inhibition of Treg migration at the time of vaccination we could achieve a certain degree of protection without undesirable side effects.

A clear upregulation of IFN-γ mRNA expression levels was observed in all vaccinated/challenged groups when compared to challenged control animals. In general, an inverse correlation was observed between the bacterial load and IFN-γ expression levels. In non-immunized/*H*. *suis*-infected mice, a persistent *H*. *suis* infection does not induce a Th1 response [29]. This indicates that immunization-evoked production of IFN-γ, a signature Th1 response cytokine, most likely plays a role in suppression and clearance of *H*. *suis*.

The majority of the immunization protocols that induced good protection against infection with *H*. *suis*, showed a clear upregulation of IL-17 mRNA expression levels. Delyria et al. showed that IL-17 expression induces the production of KC, MIP-2 and LIX primarily by murine fibroblasts and gastric epithelial cells [39]. In the present study, this relationship was confirmed in subcutaneously immunized animals and in particular with Freund’s adjuvants. Although a marked upregulation of IL-17 mRNA expression levels was also observed in the group intranasally immunized with lysate and CT, this was not accompanied by an upregulation of mRNA expression levels of KC, MIP-2 and LIX. Also, despite imparting protection, subcutaneous vaccination with *H*. *suis* lysate adjuvanted with the CCR4 antagonist did not induce Th17 responses. Therefore, the role of IL-17 in the protection against *H*. *suis* infection requires further investigation (Figs 8 and 9).

The major side-effects concerning vaccination against *H*. *suis* were found to be post-vaccination gastritis and increased mortality rates, especially when using CT-based vaccination protocols. In this study we aimed at reducing these side effects. In all immunization protocols using CT as an adjuvant, an increased vaccination/challenge-related mortality was observed as compared to other adjuvants. Four mice that were immunized intranasally with CT as adjuvant in the first study, died within 48 hours after *H*. *suis* challenge, most likely because of a pneumonia, which was confirmed histopathologically. In the second study, two mice also died in each group immunized with CT (IN and SL), within 24 hours after challenge with *H*. *suis*, most likely of an immunization/challenge related pneumonia, despite drastically lowering the volume of the vaccines. Histopathological evaluation showed the presence of oedema in the interstitial space of the lungs of these mice. In the other groups, the mortality rates were lower.

In all subcutaneously immunized/challenged groups of the first study, severe post-vaccination gastritis and pseudo-pyloric metaplasia of the stomach epithelium were observed. In the second study, pseudo-pyloric metaplasia was most pronounced in the groups immunized with CT/lysate/SL and FC/lysate/SC. Interestingly, in the CCR4 antagonist/lysate/SC immunized animals, showing a similar level of protection, this pseudo-pyloric metaplasia was shown to be less severe and sometimes even absent. This clearly warrants further studies with CCR4 antagonists as adjuvant, aiming at further optimization of immunization protocols. Once further optimized in rodent models, these studies should also be performed in pigs, which are the natural hosts of *H*. *suis*. Although CT was shown to induce substantial side effects in mice in the present study and although CT is known to be enterotoxic in humans, Cox et al. describe less toxicity in pigs, the natural host of *H*. *suis*. In 3 to 4 week old piglets, a dose of 1mg of CT induced only a pasty to semiliquid diarrhea for 2h [28]. A major focus for further research should be the use of non-toxic derivates of CT that still retain significant adjuvanticity. In addition, future research should be performed on the potential use of CCR4 antagonists in pig vaccination.

In conclusion, subcutaneous immunization protocols with FC/lysate were shown to confer an equally good protection against subsequent *H*. *suis* challenge compared to the previously used CT/lysate intranasal immunization protocols, although a severe pseudo-pyloric metaplasia was observed as well. Interestingly, a newly developed CCR4 antagonist was shown to induce similar levels of protection when used as a vaccine adjuvant for subcutaneous vaccination, whilst clearly triggering less side effects. Finally, the vaccination protocols that showed good results in the current rodent model should be confirmed in a pig model.

## References

[1] Hellemans A, Chiers K, Maes D, De Bock M, Decostere A, Haesebrouck F, et al (2007) Prevalence of ‘*Candidatus* Helicobacter suis’ in pigs of different ages. Veterinary record. 161: 189–192. 17693628 10.1136/vr.161.6.189

[2] De Bruyne E, Flahou B, Chiers K, Meyns T, Kumar S, Vermoote M, et al (2012) An experimental *Helicobacter suis* infection causes gastritis and reduced daily weight gain in pigs. Veterinary Microbiology. 160: 449–544. 10.1016/j.vetmic.2012.06.031 22776514

[3] Haesebrouck F, Pasmans F, Flahou B, Chiers K, Baele M, Meyns T, et al (2009) Gastric *Helicobacters* in domestic animals and non-human primates and their significance for human health. Clinical Microbiology Reviews. 22: 203–223. 10.1128/CMR.00041-08 PMC2668234 19366912

[4] Blanchard TG, Nedrud JG. (2010) *Helicobacter pylori* vaccines In: Sutton P, Mitchell HM (eds.), *Helicobacter pylori* in the 21^st^ century CAB International, Oxfordshire, UK, pp. 167–189.

[5] Davies MN, Bayry J, Tchilian EZ, Vani J, Shaila MS, Forbes EK, et al. (2009) Toward the Discovery of Vaccine Adjuvants: Coupling *In Silico* Screening and *In Vitro* Analysis of Antagonist Binding to Human and Mouse CCR4 Receptors. PLoS One. 10.1371/journal.pone.0008084 PMC2787246 20011659

[6] Corthésy-Theulaz I, Porta N, Glauser M, Saraga E, Vaney AC, Haas R, et al (1995) Oral immunization with *Helicobacter pylori* urease B subunit as a treatment against *Helicobacter* infection in mice. Gastroenterology. 109: 115–121. 7797009 10.1016/0016-5085(95)90275-9

[7] Banerjee S, Medina Fatimi A, Nichols R, Tendler D, Michetti M, Simon J, et al (2002) Safety and efficacy of low dose *Escherichia coli* enterotoxin adjuvant for urease based oral immunization against *Helicobacter pylori* in healthy volunteers,” Gut. 51: 634–640. 12377799 10.1136/gut.51.5.634 PMC1773429

[8] Garhart CA, Redline RW, Nedrud JG, Czinn SJ. (2002) Clearance of *Helicobacter pylori* infection and resolution of post-immunization gastritis in a kinetic study of prophylactically immunized mice. Infection and Immunity. 70: 3529–3538. 12065492 10.1128/IAI.70.7.3529-3538.2002 PMC128038

[9] Guy B, Hessler C, Fourage S, Haensler J, Vialon-Lafay E, Robki B, et al (1998) Systemic immunization with urease protects mice against *Helicobacter pylori* infection. Vaccine. 16: 850–856. 9627943 10.1016/s0264-410x(97)00258-2

[10] Guy B, Hessler C, Fourage S, Robki B, Quentin Millet MJ. (1999) Comparison between targeted and untargeted systemic immunizations with adjuvanted urease to cure *Helicobacter pylori* infection in mice. Vaccine. 17: 1130–1135. 10195624 10.1016/s0264-410x(98)00332-6

[11] Nyström J, Raghavan S, Svennerholm AM. (2006) Mucosal immune responses are related to the reduction of bacterial colonization in the stomach after therapeutic *Helicobacter pylori* immunization in mice. Microbes and Infection. 8: 442–449. 16243563 10.1016/j.micinf.2005.07.010

[12] Sutton P, Doidge C, Pinczower G, Wilson J, Harbour S, Swierczak A, et al (2007) Effectiveness of vaccination with recombinant HpaA from *Helicobacter pylori* is influenced by host genetic background. FEMS Immunology and Medical Microbiology. 50: 213–219. 17567282 10.1111/j.1574-695X.2006.00206.x

[13] Svennerholm AM, Lundgren A. (2007) Progress in vaccine development against *Helicobacter pylori* . FEMS Immunology and Medical Microbiology. 50: 146–156. 17442014 10.1111/j.1574-695X.2007.00237.x

[14] Xie Y, Zhou NJ, Gong YF, Zhou XJ, Chen J, Hu SJ, et al (2007) The immune response induced by *Helicobacter pylori* vaccine with chitosan as adjuvant and its relation to immune protection. World Journal of Gastroenterology. 13: 1547–1553. 17461447 10.3748/wjg.v13.i10.1547 PMC4146897

[15] Jiang W, Baker HJ, Smith BF. (2003) Mucosal immunization with *Helicobacter*, CpG DNA, and cholera toxin is protective. Infection and Immunity. 71: 40–46. 12496147 10.1128/IAI.71.1.40-46.2003 PMC143171

[16] Raghavan S, Nyström J, Frederiksson M, Holmgren J, Harandi AM. (2003) Orally administered CpG oligodeoxynucleotide induces production of CXC and CC chemokines in the gastric mucosa and suppresses bacterial colonization in a mouse model of *Helicobacter pylori* infection. Infection and Immunity. 71: 7014–7022. 14638791 10.1128/IAI.71.12.7014-7022.2003 PMC308895

[17] Anderl F, Gerhard M. (2014) *Helicobacter pylori* vaccination: Is there a path to protection? World Journal of Gastroenterology 34: 11939–11949. 10.1371/journal.pone.0008084 PMC4161780 25232229

[18] Flahou B, Hellemans A, Meyns T, Duchateau L, Chiers K, Baele M, et al (2009) Protective immunization with homologous and heterologous antigens against *Helicobacter suis* challenge in a mouse model. Vaccine. 27: 1416–1421. 10.1016/j.vaccine.2008.12.031 19136039

[19] Vermoote M, Flahou B, Pasmans F, Ducatelle R, Haesebrouck F (2013) Protective efficacy of vaccines based on the *Helicobacter suis* urease subunit B and γ-glutamyl transpeptidase. Vaccine. 10.1016/j.vaccine.2013.05.047 23707444

[20] Bayry J, Tartour E, Tough DF. (2014) Targeting CCR4 as an emerging strategy for cancer therapy and vaccines. Trends Pharmacol Sci. 35:163–165. 10.1016/j.tips.2014.02.003 24612600

[21] Bayry J, Tchilian EZ, Davies MN, Forbes EK, Draper SJ, Kaveri SV, et al (2008) In silico identified CCR4 antagonists target regulatory T cells and exert adjuvant activity in vaccination. Proc Natl Acad Sci. USA 105: 10221–10226. 10.1073/pnas.0803453105 18621704 PMC2481334

[22] Sather BD, Treuting P, Perdue N, Miazgowicz M, Fontenot JD, Rudensky AY, et al (2007) Altering the distribution of Foxp3(+) regulatory T cells results in tissue-specific inflammatory disease. J Exp Med. 204: 1335–147. 17548521 10.1084/jem.20070081 PMC2118615

[23] Bonecchi R, Bianchi G, Bordignon PP, D'Ambrosio D, Lang R, Borsatti A, et al (1998) Differential expression of chemokine receptors and chemotactic responsiveness of type 1 T helper cells (Th1s) and Th2s. J Exp Med. 187: 129–134. 9419219 10.1084/jem.187.1.129 PMC2199181

[24] André S, Tough DF, Lacroix-Desmazes S, Kaveri SV, Bayry J. (2009) Surveillance of antigen-presenting cells by CD4+ CD25+ regulatory T cells in autoimmunity: immunopathogenesis and therapeutic implications. Am J Pathol. 174(5):1575–87 10.2353/ajpath.2009.080987 19349365 PMC2671245

[25] Pere H, Montier Y, Bayry J, Quintin-Colonna F, Merillon N, Dransart E, Badoual C, et al (2011) A CCR4 antagonist combined with vaccines induces antigen-specific CD8+ T cells and tumor immunity against self antigens. Blood 118:4853–62. 10.1182/blood-2011-01-329656 21908423

[26] Baele M, Decostere A, Vandamme P, Ceelen L, Hellemans A, Chiers K, et al (2008) Isolation and characterization of *Helicobacter suis* sp. nov. from pig stomachs. International Journal of Systematic and evolutionary Microbiology. 58: 1350–1358. 10.1099/ijs.0.65133-0 18523177

[27] Higashi T, Hashimoto K, Takagi R, Mizuno Y, Okazaki Y, Tanaka Y, et al (2010) Curdlan induces DC-mediated Th17 polarization via Jagged1 activation in human dendritic cells. Allergol Int. 59(2):161–6. 10.2332/allergolint.09-OA-0103 20179419

[28] Cox JC, Coulter AR. (1997) Adjuvants—a classification and review of their modes of action. Vaccine. 15(3):248–56. 9139482 10.1016/s0264-410x(96)00183-1

[29] Flahou B, Van Deun K, Pasmans F, Smet A, Volf J, Rychlik I, et al (2012) The local immune response of mice after *Helicobacter suis* infection: strain differences and distinction with *Helicobacter pylori* . Veterinary Research. 43:75 10.1186/1297-9716-43-75 23107128 PMC3537685

[30] O'Rourke JL, Solnick JV, Neilan BA. (2004) Description of ‘Candidatus Helicobacter heilmannii’ based on DNA sequence analysis of 16S rRNA and urease genes. Int J Syst Evol Microbiol. 54: 2203–11. 15545459 10.1099/ijs.0.63117-0

[31] Livak KJ, Schmittgen TD. (2001) Analysis of relative gene expression data using real-time quantitative PCR and the 2^−ΔΔCT^ method. Methods. 25: 402–408. 11846609 10.1006/meth.2001.1262

[32] Stolte M, Meining A. (2001) The updated Sydney system: classification and grading of gastritis as the basis of diagnosis and treatment. Can J Gastroenterol. 15(9):591–8. Review. 11573102 10.1155/2001/367832

[33] Ermak TH, Giannasca PJ, Nichols R, Meyers GA, Nedrud J, Weltzin R, et al (1998) Immunization of mice with urease vaccine affords protection against *Helicobacter pylori* infection in the absence of antibodies and is mediated by MHC class-II restricted responses. The Journal of Experimental Medicine. 188: 2277–2288. 9858514 10.1084/jem.188.12.2277 PMC2212427

[34] Goto T, Nishizono A, Fujioka T, Ikewaki J, Mifune K, Nasu M. (1999) Local secretory immunoglobulin A and post-immunization gastritis correlate with protection against *Helicobacter pylori* infection after oral vaccination of mice. Infection and Immunity. 67: 2531–2539. 10225917 10.1128/iai.67.5.2531-2539.1999 PMC116000

[35] Morihara F, Fujii R, Hifumi E, Nishizono A, Uda T. (2007) Effects of vaccination by a recombinant antigen ureB 138 (a segment of the β-subunit of urease) against *Helicobacter pylori* infection. Journal of Medical Microbiology. 56: 847–853. 17510273 10.1099/jmm.0.47061-0

[36] Kao JK, Zhang M, Miller MJ, Mills JC, Wang B, Liu M, et al (2010) *Helicobacter pylori* escape is mediated by dendritic cell-induced Treg skewing and Th17 suppression in mice,” Gastroenterology. 138: 1046–1054. 10.1053/j.gastro.2009.11.043 19931266 PMC2831148

[37] Ismail HF, Fick P, Zhang J, Lynch RG, Berg DJ. (2003) Depletion of neutrophils in IL-10^-/-^ mice delays clearance of gastric *Helicobacter* infection and decreases the Th1 immune response to *Helicobacter* . Journal of Immunology. 170: 3782–3789. 10.4049/jimmunol.170.7.3782 12646644

[38] Matsumoto Y, Blanchard TG, Drakes ML, Basu M, Redline RW, Levine AD, et al (2005) Eradication of *Helicobacter pylori* and resolution of gastritis in the gastric mucosa of Il-10-deficient mice. Helicobacter. 10: 407–415. 16181351 10.1111/j.1523-5378.2005.00349.x

[39] DeLyria ES, Redline RW, Blanchard TG. (2009) Vaccination of mice against *H*. *pylori* induces a strong Th-17 response and immunity that is neutrophil dependent. Gastroenterology. 136: 247–256. 10.1053/j.gastro.2008.09.017 18948106 PMC4960660

